# Imidacloprid-Induced Ferroptosis-Associated Injury in Common Carp Hepatocytes: Potential Involvement of the miR-153c/SQSTM1 Axis

**DOI:** 10.3390/toxics14090814

**Published:** 2026-09-13

**Authors:** Huijie Chen, Yitong Wang, Zhenkai Yao, Jing Li, Peng Li, Lei Diao

**Affiliations:** 1 School of Biological and Pharmaceutical Engineering, Jilin Agricultural Science and Technology College, 77 Hanlin Road, Jilin 132101, China; 2 Vaccine Inspection Department, Jilin Provincial Drug Inspection and Verification Center, No. 657 Zhanjiang Road, Economic and Technological Development Zone, Changchun 130033, China; 3 Drug and Cosmetics Inspection Laboratory, Inspection and Testing Center, Jiyuan Industry-City Integration Demonstration Zone, No. 181 North Wenchang Road, Jiyuan 459000, China

**Keywords:** imidacloprid, mitochondrial dysfunction, ferroptosis, common carp hepatocytes

## Abstract

The neonicotinoid insecticide imidacloprid (IMD), widely used in agriculture, poses potential risks to aquatic ecosystems, yet its hepatotoxic mechanisms in freshwater fish remain poorly understood. This study investigates the roles of ferroptosis, mitochondrial dysfunction, inflammation, and the miR-153c/SQSTM1-mediated ferritinophagy pathway in IMD-induced hepatotoxicity in carp (*Cyprinus carpio*) hepatocytes. Using the CCK-8 assay, 0.6 μM IMD was selected for subsequent experiments. IMD exposure triggered ferroptosis, evidenced by increased intracellular Fe^2+^ accumulation, upregulated pro-ferroptotic gene and protein expression, and downregulated ferroptosis inhibitors (SLC7A11 and GPX4). Mitochondrial dysfunction was confirmed by reduced ATP content, decreased mtDNA levels, and a lowered NADPH/NADP^+^ ratio, along with aberrant expression of mitochondrial fission (Fis1 and Drp1) and fusion (Mfn1 and TFAM) genes. Additionally, IMD induced oxidative stress (elevated ROS and MDA; decreased T-AOC, CAT, and SOD activities) and a pronounced inflammatory response with upregulated pro-inflammatory cytokines. Mechanistically, IMD downregulated miR-153c, with concomitant upregulation of SQSTM1 at both the mRNA and protein levels, and dual-luciferase assays validated SQSTM1 as a direct target of miR-153c. miR-153c overexpression attenuated IMD-induced ferritinophagy, ferroptosis, and inflammation via the miR-153c/SQSTM1 axis. Collectively, these findings demonstrate that IMD induces hepatotoxicity in carp by triggering ferroptosis, mitochondrial dysfunction, and inflammation through the miR-153c/SQSTM1-mediated ferritinophagy pathway, providing mechanistic insights and potential targets for mitigating IMD’s ecological risks.

## 1. Introduction

Imidacloprid (IMD, IUPAC name: (E)-1-[(6-Chloro-3-pyridinyl)methyl]-N-nitro-2-imidazolidinimine) is a broad-spectrum neonicotinoid insecticide extensively used in global agriculture [1]. IMD is detectable in various natural environments due to its prolonged half-life and high residual concentrations [2]. Its high water solubility and environmental persistence lead to frequent detection in diverse aquatic systems, with concentrations reaching 157.5 μg/L [3,4]. A global review of 29 monitoring studies across nine countries reported geometric mean imidacloprid concentrations of 0.13 μg/L in surface waters, with maximum concentrations reaching 0.63 μg/L, and 81% of the surveyed sites exceeding the proposed acute ecological threshold of 0.2 μg/L [5]. In river systems, imidacloprid concentrations ranging from 0.6 to 104.5 μg/L have been detected, with 93% of samples containing at least two neonicotinoids [6]. In contrast, concentrations in paddy fields and ponds can be substantially higher, with peak imidacloprid concentrations reaching 152 μg/L in paddy water following agricultural application [7]. This establishes IMD as a significant pollutant in aquatic ecosystems. Previous studies indicate that IMD exhibits multiple toxic effects in fish, ranging from acute lethality to chronic sublethal impacts, posing considerable ecological risks. Under long-term exposure to sublethal concentrations, IMD can bioaccumulate in fish, triggering a series of complex pathological effects [8,9,10]. The liver, a central organ for metabolism and detoxification in fish, represents a primary target for IMD-induced toxicity [11,12]. IMD could induce hepatic oxidative stress and alter the activity of detoxification enzymes in zebrafish [11,13]. Furthermore, its accumulation in fish bodies suggested that prolonged exposure may exacerbate liver damage through multiple mechanisms [14,15]. Although studies on the hepatotoxicity of IMD in fish are limited, its established toxicity and endocrine-disrupting effects in aquatic organisms imply that IMD likely adversely affects the fish liver, warranting further investigation.

Ferroptosis, a regulated cell death process driven by iron and defined by glutathione deficiency and profound lipid peroxidation, is now recognized as a pivotal player in organ damage resulting from xenobiotic exposure [16]. This process is tightly regulated by a network of molecules, including GPX4 and SLC7A11, which maintain redox balance [17]. Ferroptosis is closely linked to mitochondrial dysfunction, which is an upstream driver that exacerbates iron overload and lipid peroxidation via impaired energy metabolism and reactive oxygen species (ROS) burst [18]. Recent studies in mammalian models have implicated ferroptosis in pesticide-induced hepatotoxicity, but its role in fish hepatocytes exposed to IMD, along with the upstream regulatory pathways, remains unknown. Elucidation of this mechanism is essential for a profound understanding of the hepatotoxic nature of IMD.

Ferritinophagy serves as a pivotal bridge linking cellular autophagy and iron metabolism. Ferritinophagy is a selective autophagic process that delivers the iron-storage protein ferritin heavy chain 1 (FTH1) to lysosomes for degradation, resulting in a surge of intracellular labile iron that fuels ferroptosis. NCOA4 is the canonical cargo receptor that directly binds FTH1 to autophagosomes [19,20]. In parallel, SQSTM1, a prototypical selective autophagy receptor containing an LC3-interacting region (LIR) and a ubiquitin-associated (UBA) domain, can also mediate ferritinophagy by recognizing ubiquitinated FTH1 and bridging it to LC3-positive autophagosomal membranes [21,22]. These two receptors employ complementary recognition mechanisms and may function cooperatively to promote FTH1 degradation under stress conditions [23]. As post-transcriptional regulators, microRNAs (miRNAs) are increasingly recognized as master switches governing the crosstalk between ferroptosis and autophagy, and their roles in modulating cellular responses to environmental toxicant stress have become progressively prominent [24]. Among these regulatory miRNAs, the functions of miR-153c in cellular metabolism and stress responses have only just begun to be elucidated. In our preliminary studies, we found that IMD exposure modulated the level of miR-153c, and we also predicted SQSTM1 as a putative target gene of miR-153c. miR-153c is an evolutionarily conserved miRNA that regulates cell death and inflammation in mammalian models; however, its role in targeting SQSTM1 to mediate ferritinophagy and ferroptosis in fish under environmental stress remains poorly defined. Furthermore, ferroptosis is inherently immunogenic because lipid peroxidation products can trigger inflammatory cascades [25,26], whereas the interplay between miR-153c/SQSTM1-mediated ferritinophagy, ferroptosis and inflammation induced by IMD exposure in fish hepatocytes is entirely uncharacterized.

Using hepatocytes of carp (*Cyprinus carpio*) as the experimental model, our investigation sought to elucidate the novel mechanistic basis of IMD-induced hepatotoxicity. We hypothesized that IMD may downregulate the expression of miR-153c, thereby relieving its post-transcriptional repression of the target gene SQSTM1 and consequently activating the SQSTM1-mediated ferritinophagic pathway. Activation of this pathway leads to FTH1 degradation and iron overload in hepatocytes, which in turn triggers ferroptosis characterized by mitochondrial dysfunction and excessive lipid peroxidation. We further examined whether IMD concurrently elicits an inflammatory response in this model. For the first time, this study systematically identifies the central regulatory role of the miR-153c/SQSTM1/ferritinophagy axis in IMD-induced ferroptosis in fish hepatocytes. This finding not only provides a novel molecular perspective and theoretical basis for the comprehensive assessment of the environmental and ecological risks posed by neonicotinoid insecticides, but also lays a critical scientific foundation for the development of detoxification intervention strategies targeting this regulatory pathway.

## 2. Materials and Methods

### 2.1. Primary Hepatocyte Isolation, Culture, and Cryopreservation

Primary hepatocytes, rather than an established cell line, were used in this study. Healthy common carp (*Cyprinus carpio*) weighing 500 ± 50 g were obtained from a local fish farm in Jilin, China, and acclimated in aerated freshwater at 25 °C for 2 weeks before the experiment. All animal procedures were approved by Jilin Agricultural Science and Technology College Experimental Animal Ethics Committee (Approval No. LL5C202503019, 10 January 2025) and performed in accordance with the Guidelines for the Care and Use of Laboratory Animals.

Hepatocytes were isolated by a modified two-step collagenase perfusion method. Briefly, fish were anesthetized with 100 mg/L of MS-222 (Sigma-Aldrich, St. Louis, MO, USA), and the liver was aseptically dissected, rinsed with ice-cold phosphate-buffered saline (PBS) to remove blood, and minced into small pieces. The tissue fragments were digested with 0.1% type IV collagenase (Sigma-Aldrich, St. Louis, MO, USA) at 25 °C for 20 min with gentle agitation. The resulting cell suspension was filtered through a 100-μm cell strainer and centrifuged at 500× *g* for 5 min at 4 °C. The cell pellet was resuspended and purified by discontinuous Percoll gradient (Sigma-Aldrich, St. Louis, MO, USA) centrifugation. The purified hepatocytes were washed twice with M199 medium and resuspended in M199 complete medium (SH30253.01, HyClone, Logan, UT, USA) supplemented with 10% fetal bovine serum (FBS), 100 U/mL of penicillin, and 100 μg/mL of streptomycin. Cell viability was assessed by trypan blue exclusion and was consistently >90%.

For cryopreservation, freshly isolated hepatocytes were suspended in freezing medium (M199 complete medium containing 10% DMSO and 20% FBS) at a density of 1 × 10^7^ cells/mL, transferred to cryovials, and stored in liquid nitrogen. For each experiment, cryopreserved hepatocytes were rapidly thawed in a 37 °C water bath under gentle agitation. The cell suspension was immediately diluted in 2 mL of M199 complete medium and centrifuged (1000 rpm, 5 min). The cell pellet was resuspended in 2 mL of fresh medium and plated in a 25-cm^2^ flask at a density of 1 × 10^6^ cells/mL, and the total volume was adjusted to 4 mL. After a 48-h recovery period in a 25 °C, 5% CO_2_ incubator, the adherent primary hepatocytes were detached with 0.05% trypsin-EDTA (WLA080a, Wanleibio, Shenyang, China) and re-plated at the appropriate density for each experiment. All experiments were performed using primary, non-immortalized hepatocytes within one passage after thawing, and no established fish hepatocyte cell line was used. To account for donor-to-donor variability, hepatocytes isolated from eight individual fish were used as independent biological replicates (*n* = 8), with each fish serving as one biological replicate.

### 2.2. Hepatocytes Viability

To assess cell viability, a CCK-8 kit (Dojindo, Kumamoto, Japan) was employed on hepatocytes from common carp (*Cyprinus carpio*). Hepatocytes were plated in 96-well plates at 1.5 × 10^5^ cells/mL, with approximately 200 μL of cell suspension per well, and allowed to adhere during a 24-h culture period. The test compound, IMD (CAS 138261-41-3, >99.98% pure), was sourced from Aladdin Reagent Co., Ltd. in Shanghai, China. After the initial incubation, the cells were exposed to various concentrations of IMD (0, 0.001, 0.01, 0.1, 0.6, 1, 10, 25, 50, 100, and 200 μM) for 24 h. The final concentration of dimethyl sulfoxide (DMSO; Cat. No.: L8665; Kerina Biotechnology Co., Ltd., Beijing, China) in the working solution was maintained below 0.01% (*v*/*v*) to avoid solvent-induced cytotoxicity. We treated the control group with DMSO alone under the same conditions. Subsequently, 3 μL of CCK-8 solution was introduced into every well. The plates were incubated for 80 min in a humidified incubator at 28 °C (optimal temperature for carp hepatocyte culture). Finally, the absorbance was measured at the appropriate wavelength (typically 450 nm) using a microplate reader, and cell viability was calculated relative to that of the control group.

### 2.3. Ferrous Iron (Fe^2+^) Fluorescent Staining

After treatments, hepatocytes were washed twice with pre-warmed PBS. The cells were then incubated with FerroOrange (Cat# MA0649, Meilun Biotechnology Co., Ltd., Dalian, China), a selective fluorescent probe for labile Fe^2+^, at a final concentration of 1 μmol/L in serum-free culture medium at 25 °C for 30 min. After incubation, the staining solution was replaced with fresh culture medium. Hepatocytes were immediately visualized using the same microscope, objective lens, exposure time, gain, and illumination intensity for all experimental groups, with appropriate filter sets for FerroOrange detection (excitation/emission: 540–550 nm/570–580 nm). Orange-red fluorescence indicated the presence of Fe^2+^. Hepatocytes were immediately visualized using the same microscope, objective lens, exposure time, gain, and illumination intensity for all experimental groups, with appropriate filter sets for FerroOrange detection (excitation/emission: 540–550 nm/570–580 nm). Fluorescence intensity was objectively quantified using ImageJ v1.53t (National Institutes of Health, Bethesda, MD, USA). For each well, five randomly selected non-overlapping fields were analyzed, and three replicate wells were included for each group. The integrated fluorescence density of each field was measured after background subtraction and normalized to the number of cells in the same field. The normalized fluorescence intensity was then expressed relative to the control group.

### 2.4. Precise Docking Prediction of RNA-Protein Binding Sites (RPISeq)

To explore the relationship between dre-miR-153c and SQSTM1, the interaction probability generated by RPISeq was retrieved via TargetScanFish (https://www.targetscan.org/fish_62/, accessed on 15 April 2025), a specialized platform for predicting common carp miRNA targets by identifying 8mer and 7mer sites matching the miRNA seed region. The resulting RPISeq probability score suggested a likely interaction between dre-miR-153c and SQSTM1, a prediction that was robust when the classifiers were validated on independent, blind datasets. Cross-validation evaluations were performed on benchmark datasets using the preset threshold, and the accuracy of the classifiers ranged from 85% to 95%.

### 2.5. Transfection of Dre-miR-153c Mimic and Dre-miR-153c Mimic NC

To investigate the regulatory function of dre-miR-153c in carp hepatocytes, dre-miR-153c mimics and negative control (mimic NC) were chemically synthesized and modified by Shanghai GenePharma Co., Ltd., Shanghai, China, with the following sequences: 5′-AUCAUGGGCUUAAUGCAAA-3′ (mimic) and 5′-AUCAUGGGCUUAAUGAAUAAAA-3′ (NC, where “m” indicates 2′-O-methylation). Hepatocytes were plated in 6-well plates at an appropriate density and transfected at approximately 85% confluency using Lipofectamine 2000 reagent (Invitrogen, Waltham, MA, USA) according to the manufacturer’s protocol. Briefly, to determine the optimal transfection dose, hepatocytes were transfected with miR-153c mimic at 50, 100, and 150 nM, and miR-153c expression was verified by qRT-PCR at 24 h post-transfection. Based on the dose-dependent upregulation of miR-153c and significant knockdown of SQSTM1, 50 nM was selected as the working dose (M50). Three experimental groups were then set up: (1) the control group (NC group), which was transfected with Lipofectamine 2000 reagent and Opti-MEM minimal essential medium (without any RNA oligonucleotides); (2) the mimic NC group, transfected with 50 nM negative control mimic using Lipofectamine 2000 and Opti-MEM; and (3) the miR-153c mimic group (M50 group), transfected with 50 nM dre-miR-153c mimic via the same transfection system. At 24 h post-transfection, hepatocytes were harvested and processed for subsequent analyses. We assessed SQSTM1 expression by measuring its protein abundance via Western blot and its mRNA transcripts via qRT-PCR. Five experimental groups were used in this study: (1) CON, untreated control hepatocytes cultured in complete medium without IMD exposure or transfection; (2) NC, transfection control hepatocytes treated with Lipofectamine 2000 and Opti-MEM in the absence of RNA oligonucleotides and IMD; (3) M50, hepatocytes transfected with 50 nM of dre-miR-153c mimic in the absence of IMD; (4) IMD, hepatocytes exposed to 0.6 μM of IMD for 24 h; and (5) IMD + M50, hepatocytes transfected with 50 nM of dre-miR-153c mimic for 6 h, followed by co-exposure to 0.6 μM of IMD for an additional 24 h.

### 2.6. Dual-Luciferase Reporter Assay

To verify the direct targeting of SQSTM1 by dre-miR-153c, a dual-luciferase reporter assay was performed. The 3′-untranslated region (3′-UTR) fragment of the common carp (*Cyprinus carpio*) SQSTM1 gene containing the predicted dre-miR-153c binding site was amplified by PCR from carp hepatocyte cDNA and cloned downstream of the firefly luciferase coding region in the pmirGLO dual-luciferase reporter vector (Promega, Madison, WI, USA). A mutant reporter (MUT) was generated by site-directed mutagenesis, in which seven nucleotides within the seed-pairing region of the dre-miR-153c binding site were replaced to disrupt complementarity, whereas the wild-type (WT) construct retained the native sequence. Carp primary hepatocytes were seeded in 24-well plates and co-transfected at approximately 80% confluency with 200 ng of either the WT or MUT reporter plasmid together with 50 nM of dre-miR-153c mimic or mimic negative control (NC), using Lipofectamine 2000 (Invitrogen, Waltham, MA, USA) according to the manufacturer’s protocol. At 24 h post-transfection, cells were lysed, and firefly and Renilla luciferase activities were measured sequentially using the Dual-Luciferase Reporter Assay System (Promega, Madison, WI, USA) on a luminometer (Promega, Madison, WI, USA). Firefly luciferase activity was normalized to Renilla luciferase activity to control for transfection efficiency.

### 2.7. Detection of ATP in Carp Hepatocytes

After the respective treatments, hepatocytes underwent washing with PBS and were harvested via scraping. The cell pellets were collected by centrifugation and lysed on ice using an appropriate lysis buffer. To remove cellular debris, the lysates were centrifuged (12,000× *g*, 10 min, 4 °C). The ATP level in the cleared supernatant was then assessed following the protocol of a commercial Fish ATP ELISA Kit (Cat# ZK-11154, Shanghai Zhen Ke Biological Technology Co., Ltd., Shanghai, China). The procedure involved adding samples to antibody-precoated wells, then introducing an HRP-conjugated detection antibody. This was followed by incubation, washing steps, and the addition of substrate for color development. Absorbance at 450 nm was measured with a microplate reader after stopping the reaction. Concentrations were interpolated from the standard curve and normalized against the total protein concentration measured in a parallel BCA protein assay.

### 2.8. Mitochondrial DNA Extraction and Quantification

After treatments, total genomic DNA was extracted from hepatocytes using a Universal Genomic DNA Extraction Kit (Cat# D2100, Solarbio, Beijing, China) according to the manufacturer’s instructions. The DNA concentration and purity were assessed using a NanoDrop spectrophotometer (Thermo Fisher Scientific, Waltham, MA, USA). Relative mitochondrial DNA (mtDNA) copy number was determined by qPCR using species-specific primers targeting the mitochondrial cytochrome b (Cyt b) gene of *Cyprinus carpio* and the nuclear single-copy gene GAPDH. The relative mtDNA copy number was calculated as the ratio of Cyt b to GAPDH using the 2^−ΔΔCt^ method.

### 2.9. Measurement of NADPH/NADP^+^ Ratio

Following treatment, hepatocytes were rinsed with ice-cold PBS and harvested. Intracellular NADP(H) was extracted using the buffer supplied with the NADP/NADPH Assay Kit (Cat# ab65349, Abcam, Cambridge, UK) according to the manufacturer’s instructions. In brief, cell pellets were suspended in extraction buffer and centrifuged at 12,000× *g* for 10 min at 4 °C. The supernatant was divided into two aliquots. One aliquot was assayed directly to determine the total NADP(H) pool (NADP^+^ + NADPH). The second aliquot was incubated at 60 °C for 30 min to selectively decompose NADP^+^, leaving NADPH intact, and was then assayed for NADPH alone. In both assays, the NADP cycling enzyme mixture and NADPH developer were added, and absorbance was measured at 450 nm using a microplate reader. Concentrations were interpolated from a standard curve. The NADP^+^ concentration was calculated as NADP^+^ = total NADP(H) − NADPH. The NADPH/NADP^+^ ratio was then computed as NADPH divided by NADP^+^. All values were normalized to the total protein concentration determined by a BCA protein assay.

### 2.10. Measurement of Lipid Peroxidation and Antioxidant Enzymes

Following treatment, hepatocytes were collected and reserved for subsequent experimental assays. Oxidative stress markers were evaluated using the corresponding assay kits. Malondialdehyde (MDA) content was assessed with the Lipid Peroxidation MDA Kit (Cat. WLA048b, Beyotime, Shanghai, China). T-AOC was evaluated following the ABTS method using the corresponding kits (Cat. WLA049b, Beyotime, Shanghai, China). SOD activity was evaluated with the SOD Assay Kit (Cat. WLA110b, Beyotime, Shanghai, China). Catalase (CAT) activity was assayed using the Catalase Assay Kit (Cat. BC0200, Solarbio, Beijing, China), with the detection principle relying on the spectrophotometric monitoring of hydrogen peroxide decomposition. All colorimetric measurements were acquired using a microplate reader. The values of MDA content, T-AOC level, and enzymatic activities (SOD and CAT) were normalized to the total protein content of each respective sample to eliminate variations in sample loading.

### 2.11. Detection of Intracellular ROS Accumulation

After being washed twice with PBS, the carp hepatocytes were incubated with the fluorescent probe 2′,7′-dichlorodihydrofluorescein diacetate for 40 min. The intracellular fluorescence signal was then observed under a fluorescence microscope (excitation/emission: 488/525 nm). The relative fluorescence intensity was normalized to the protein concentration of the cell lysate.

### 2.12. Hepatocytes Immunofluorescence

Hepatocytes were seeded onto sterile glass coverslips placed in 24-well plates. After the respective treatments, hepatocytes were rinsed three times with PBS prior to fixation with 4% paraformaldehyde for 20 min at 25 °C. Cells were permeabilized with 0.3% Triton X-100 (Sigma-Aldrich, St. Louis, MO, USA) for 15 min at 25 °C. To block nonspecific antigen-binding sites, cells were incubated with 5% BSA in PBS for 1 h at 25 °C. For the assessment of inflammatory cytokine expression, hepatocytes were incubated overnight at 4 °C with anti-TNF-α and anti-IL-6. For assessment of ferritinophagy markers, hepatocytes were incubated overnight at 4 °C with anti-FTH1 or anti-LC3. All primary antibodies were diluted in PBS supplemented with 1% BSA at the manufacturer-recommended dilutions. Cells were then washed three times (5 min per wash) with PBS containing 0.1% Tween-20 (PBST) and incubated for 1 h at 25 °C in the dark with Cy3-conjugated goat anti-rabbit IgG or FITC-conjugated goat anti-mouse IgG (GB22301, Servicebio, Wuhan, China), as appropriate for the primary antibody host species. After three washes with PBST and one final wash with PBS, cell nuclei were stained with DAPI for 8 min. Hepatocytes were observed by laser scanning confocal microscope, and fluorescence intensity was quantified using ImageJ software from five randomly selected fields per well, with three replicate wells per group.

### 2.13. Detection of mRNA and miRNA Levels

Total RNA was extracted from hepatocytes using Trizol reagent (Invitrogen, Waltham, MA, USA). For mRNA analysis, 1 μg of total RNA was synthesized into the first strand cDNA using the primescript RT kit (Takara Bio, Kusatsu, Shiga, Japan). qRT-PCR was performed using TB Green Premix Ex Taq II (Takara Bio, Kusatsu, Shiga, Japan). For miRNA analysis, specific stem-loop reverse transcription primers were used for cDNA synthesis. The primer sequences are listed in Table S1. Target gene expression was quantified via the 2^−ΔΔCt^ method and normalized to GAPDH (mRNA) or U6 snRNA (miRNA).

### 2.14. Protein Extraction and Analysis

Hepatocytes were lysed on ice using RIPA buffer (Beyotime, Shanghai, China) with 1 mM PMSF. The lysates underwent centrifugation at 12,000× *g* for 15 min at 4 °C, and we collected the resulting supernatants. Protein concentration was determined using a BCA Protein Assay Kit (P0012S, Beyotime, Shanghai, China). Equal amounts of protein (25 μg) were separated by 10% or 12% sodium dodecyl sulfate-polyacrylamide gel electrophoresis (SDS-PAGE) and then transferred onto polyvinylidene fluoride (PVDF) membranes. The membranes were blocked with 5% non-fat milk in Tris-buffered saline containing 0.1% Tween-20 (TBST) for 1.5 h at room temperature. For primary antibody incubation, the membranes were kept at 4 °C overnight. The antibodies used and their respective dilution ratios, including the GAPDH antibody used as the internal loading control, are provided in Table S2. After incubation with the corresponding secondary antibody for 50 min, the membranes were washed three times with TBST and visualized using a chemiluminescence imaging system. ImageJ software was used to quantify the intensity of each protein band, and the abundance of each target protein was normalized to the corresponding GAPDH band from the same sample. GAPDH was used as the internal loading control for all Western blot analyses throughout the study, and no obvious treatment-related changes in GAPDH band intensity were observed among the experimental groups.

### 2.15. Statistical Analysis

Statistical analyses were performed using GraphPad Prism v9.0 (GraphPad Software, San Diego, CA, USA). Data are presented as the mean ± SD of eight independent biological replicates (*n* = 8), with hepatocytes obtained from eight individual fish. Before statistical comparisons, data normality was assessed using the Shapiro–Wilk test, and homogeneity of variance was evaluated using Levene’s test. For datasets that satisfied the assumptions of normality and homogeneity of variance, differences between two groups were analyzed using an unpaired two-tailed Student’s *t*-test, whereas comparisons among multiple groups were performed using one-way ANOVA followed by Tukey’s post hoc test. Statistical significance was defined as *p* < 0.05. All datasets analyzed using parametric tests satisfied the assumptions of normality and homogeneity of variance.

## 3. Results

### 3.1. Effects of IMD Exposure on Ferroptosis in Hepatocytes of Carp

**Figure 1:**
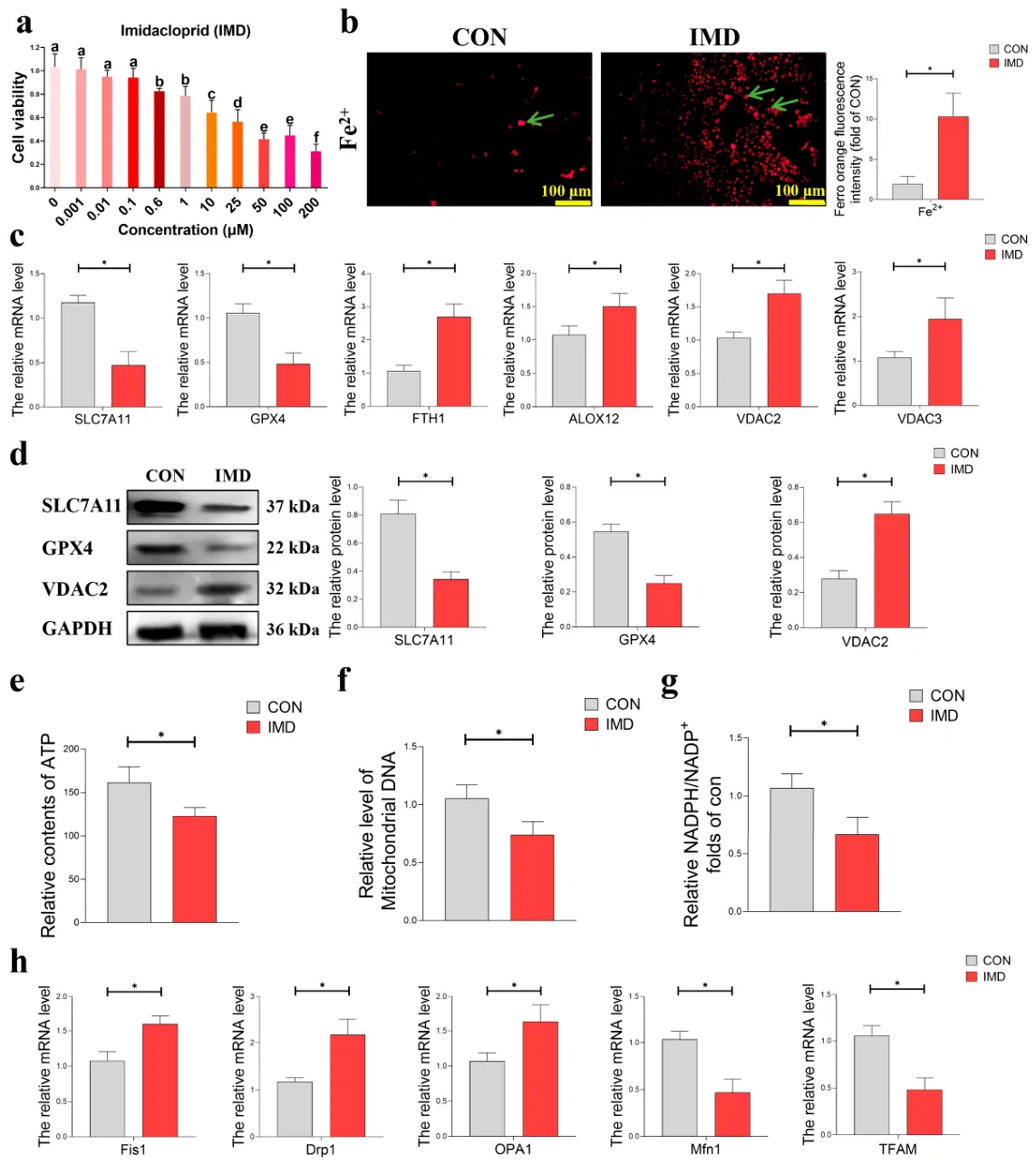
IMD-mediated ferroptosis in hepatocytes after 24 h of treatment. (**a**) The hepatocytes were exposed to IMD (0, 0.001, 0.01, 0.1, 0.6, 1, 10, 25, 50, 100 and 200 μM) in hepatocytes to detect its activity (*n* = 8; *p* < 0.05). (**b**) Representative FerroOrange staining micrographs of intracellular Fe^2+^ accumulation in hepatocytes post IMD treatment (scale bars = 100 μm, *n* = 8). The green arrow represents the Fe^2+^ positive fluorescence signal. (**c**) SLC7A11, GPX4, FTH1, ALOX12, VDAC2 and VDAC3 mRNA expression in CON and IMD groups (*n* = 8; *p* < 0.05). (**d**) GAPDH-normalized ferroptosis-associated protein abundance in CON and IMD groups (*n* = 8; *p* < 0.05). (**e**,**f**) ATP content (**e**) and DNA abundance (**f**) in CON and IMD hepatocytes (*n* = 8; *p* < 0.05). (**g**) NADPH/NADP^+^ ratio in CON and IMD groups (*n* = 8; *p* < 0.05). (**h**) Mitochondrial function-associated gene mRNA expression in CON and IMD groups (*n* = 8; *p* < 0.05). Data are presented as mean ± SD (*n* = 8). Different lowercase letters indicate significant differences (*p* < 0.05). Statistical significance was determined by an unpaired two-tailed Student’s *t*-test. * *p* < 0.05 versus the CON group.

The cytotoxic effect of IMD on hepatocytes was evaluated via the CCK-8 assay. As depicted in Figure 1a, the IC50 of IMD was 26.4 μM. Cell viability declined in a concentration-dependent manner with escalating IMD doses (0, 0.001, 0.01, 0.1, 0.6, 1, 10, 25, 50, 100 and 200 μM). A significant reduction in viability was observed at concentrations of 0.6, 1, 10, 25, 50, 100 and 200 μM (*p* < 0.05). From these data, 0.6 μM IMD was chosen for subsequent experiments, which induced significant hepatocyte death after a 24-h incubation period. These findings demonstrate that IMD effectively induces hepatotoxicity in carp (*Cyprinus carpio*) hepatocytes.

To investigate the disruption of iron homeostasis, we first assessed intracellular Fe^2+^ levels. Fe^2+^ staining revealed a pronounced accumulation of labile iron in IMD-treated hepatocytes, with fluorescence intensity increasing by approximately 331.38% versus the CON group (Figure 1b). The transcriptional levels of key ferroptosis-related genes were then examined. qRT-PCR results indicated that IMD upregulated the mRNA levels of the pro-ferroptotic genes FTH1, ALOX12, VDAC2, and VDAC3 by 157.52%, 39.4%, 64.07%, and 80.07%, respectively (Figure 1c). Conversely, the mRNA levels of the ferroptosis inhibitors SLC7A11 and GPX4 were significantly downregulated by 59.91% and 54.45%, respectively (*p* < 0.05). Consistent with the transcriptional changes, Western blot analysis confirmed the suppression of the ferroptosis defense system at the protein level. The protein abundances of SLC7A11 and GPX4 were markedly reduced by 57.5% and 54.1% in the IMD group (*p* < 0.05; Figure 1d). In parallel, the level of VDAC2 protein, which facilitates mitochondrial dysfunction, was increased by approximately 133.41% (*p* < 0.05). Collectively, these results indicated that IMD exposure led to profound dysregulation of iron homeostasis and a shift in the expression balance of ferroptosis regulators, favoring cell death execution.

Mitochondrial damage triggers increased fission and decreased fusion, leading to mitochondrial fragmentation, which in turn exacerbates ferroptosis in hepatocytes. To assess IMD’s impact on mitochondrial function in carp hepatocytes, ATP content, mtDNA levels, and the NADPH/NADP^+^ ratio were measured (Figure 1e–h). As shown in Figure 1e, relative to the CON group, IMD exposure reduced hepatocellular ATP content by 23.96% (*p* < 0.05). As shown in Figure 1f, the mtDNA content in the IMD group was reduced by 29.55% relative to the CON group (*p* < 0.05). Additionally, as shown in Figure 1g, the NADPH/NADP+ ratio in the IMD group was markedly lowered by 37.76% relative to the CON group (*p* < 0.05). Collectively, these findings confirm that IMD impairs mitochondrial function in carp hepatocytes by reducing ATP content, mtDNA level, and NADPH/NADP^+^ ratio. To further elucidate the regulatory mechanisms underlying IMD-induced mitochondrial dysfunction, qRT-PCR was performed to determine the mRNA expression levels of genes associated with mitochondrial function. Compared with the CON group, the mRNA expression levels of Fis1, Drp1, and OPA1 in the IMD group were significantly upregulated by 48.69%, 84.56%, and 52.73%, respectively (*p* < 0.05) (Figure 1h). These genes are core modulators of mitochondrial fission, suggesting that IMD exposure alters mitochondrial dynamics. Conversely, the expression of Mfn1 and TFAM (genes that facilitate mitochondrial fusion in hepatocytes) was downregulated by 54.89% and 54.45%, respectively. This observation suggests that hepatocytes mount a multifaceted cellular response encompassing mitochondrial fission and fusion events, likely representing an adaptive cellular endeavor to mitigate IMD-mediated stressors.

### 3.2. The Impact of IMD on Oxidative Stress in Hepatocytes

**Figure 2:**
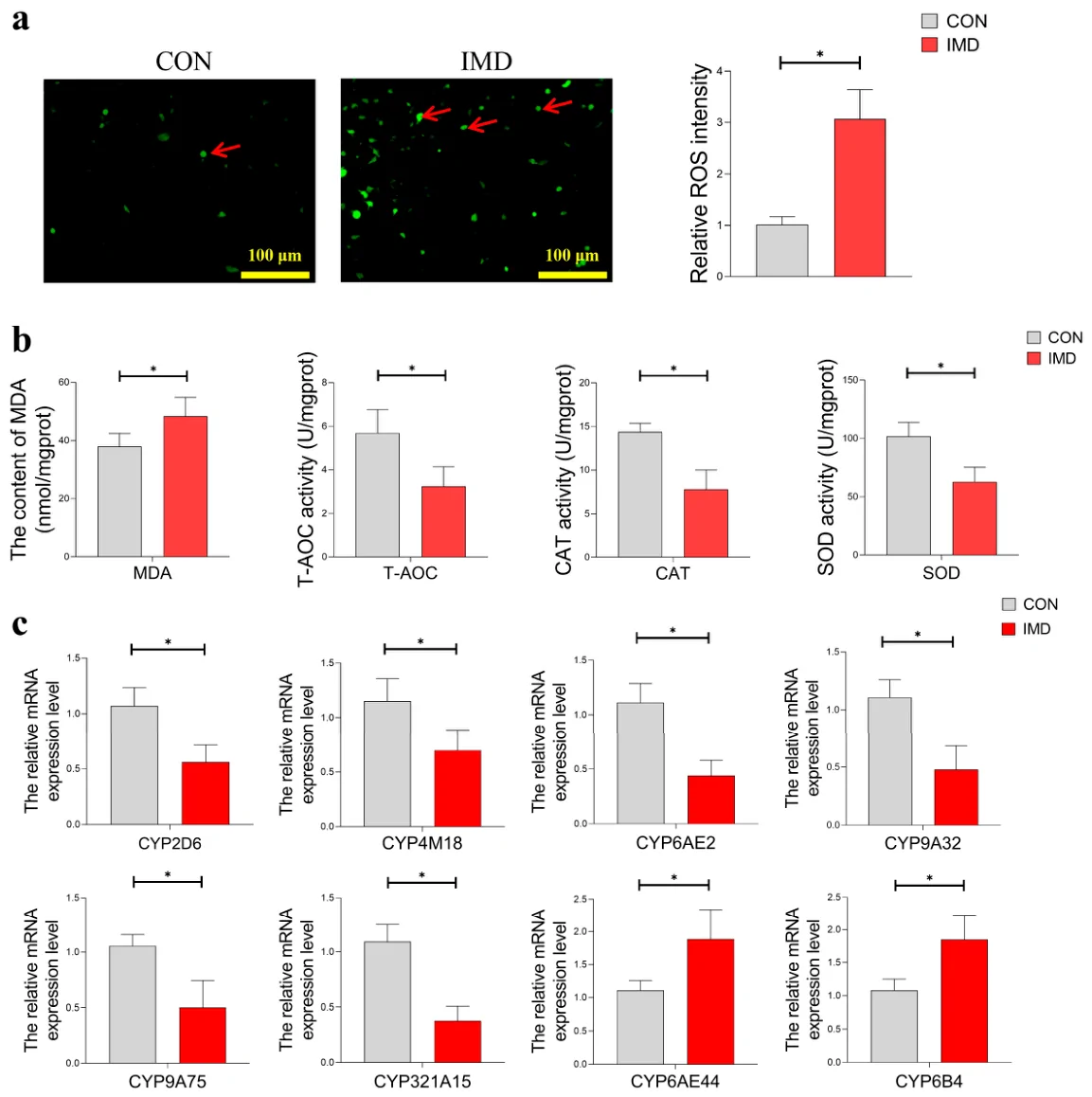
Following 24-h exposure to IMD, carp hepatocytes exhibited significant alterations in oxidative stress and ROS-xenobiotics metabolism pathway. (**a**) Intracellular ROS levels were quantified in IMD-exposed hepatocytes (*n* = 8; *p* < 0.05). The red arrow represents the ROS positive fluorescence signal. (**b**) Oxidative stress markers (MDA levels alongside T-AOC, CAT and SOD activities) were significantly changed upon IMD treatment (*n* = 8; *p* < 0.05). (**c**) mRNA expression of key xenobiotic metabolism-associated genes was significantly modulated in IMD-exposed hepatocytes versus CON group. Data are presented as mean ± SD (*n* = 8). Statistical significance was determined by an unpaired two-tailed Student’s *t*-test. * *p* < 0.05 versus the CON group.

To assess redox balance in IMD-exposed hepatocytes, ROS levels, MDA content, and T-AOC, CAT and SOD activities were assayed. Compared with the CON group, IMD exposure significantly increased ROS generation by 201.43% (Figure 2a). For MDA (Figure 2b), IMD-exposed cells exhibited a 27.51% rise in this index relative to the CON group (*p* < 0.05), while T-AOC, CAT and SOD activities were markedly reduced by 42.96%, 45.6% and 38.1% respectively (*p* < 0.05).

To elucidate the mechanism underlying the role of these genes in regulating excessive ROS, the mRNA levels of genes involved in the ROS-xenobiotics metabolism pathway were detected in hepatocytes. Figure 2c illustrates that compared with the CON group, the IMD group exhibited marked reductions in the mRNA expression levels of xenobiotic pathway-related genes, including CYP2D6 (47.64%), CYP4M18 (39.5%), CYP6AE2 (60.47%), CYP9A32 (56.74%), CYP9A75 (52.71%), and CYP321A15 (65.38%) (all *p* < 0.05). Conversely, mRNA expression of CYP6AE44 and CYP6B4 within IMD-treated hepatocytes was markedly elevated by 69.79% and 70.54%, respectively (both *p* < 0.05). Notably, these data demonstrated that IMD disrupted the xenobiotic metabolism pathway associated with ROS generation in carp hepatocytes.

### 3.3. IMD Induces Inflammatory Cytokine Secretion in Carp (Cyprinus carpio) Hepatocytes

**Figure 3:**
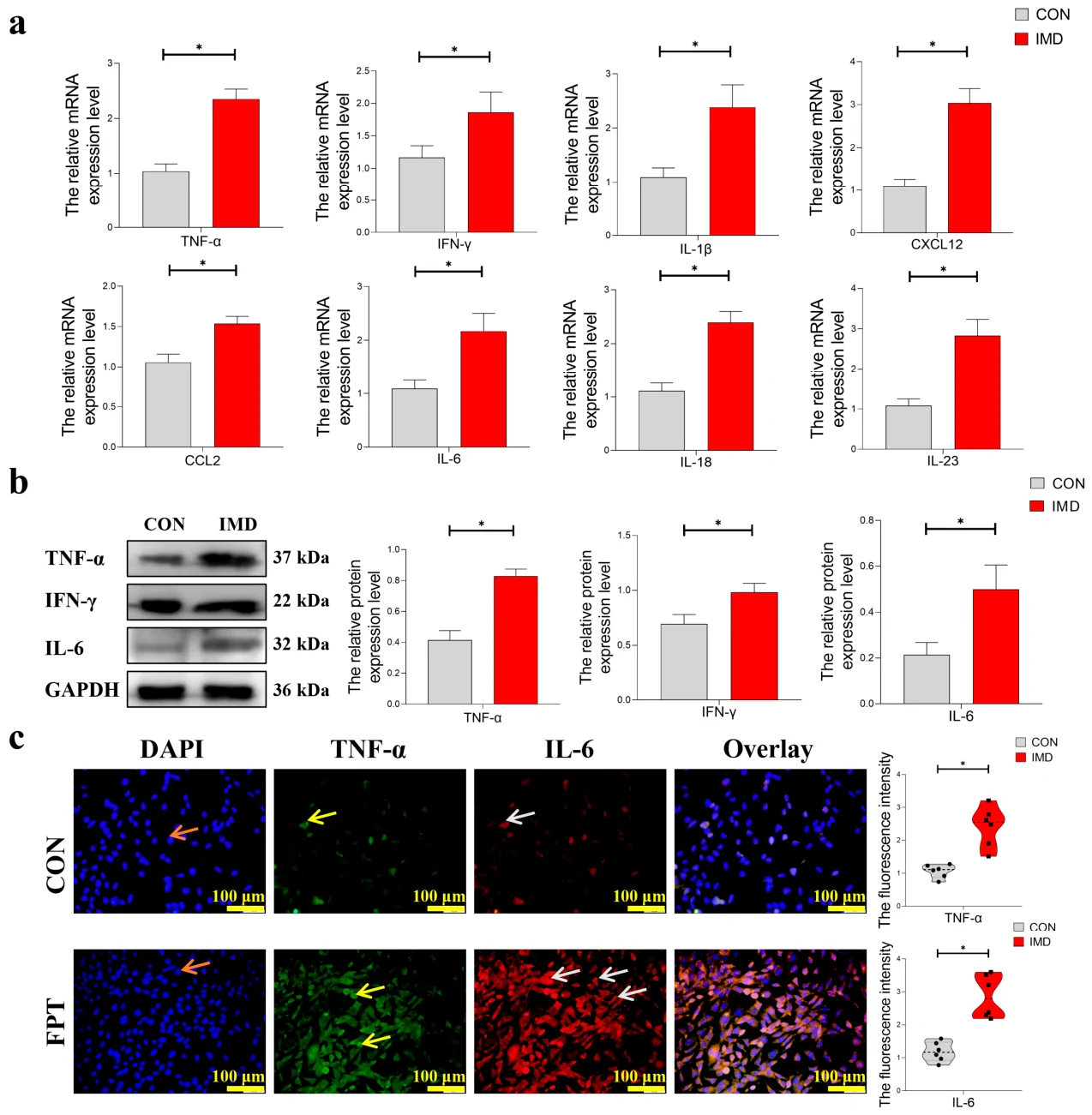
Pro-inflammatory response in carp hepatocytes following 24-h IMD exposure. (**a**) The mRNA level patterns of key inflammation in IMD-treated hepatocytes (*n* = 8; *p* < 0.05). (**b**) GAPDH-normalized protein abundances of the indicated cytokines in IMD-treated hepatocytes (*n* = 8; *p* < 0.05). (**c**) Representative immunofluorescence images of TNF-α and IL-6 staining in IMD-treated hepatocytes compared to the CON group (scale bar: 100 μm; *n* = 8; *p* < 0.05). The red arrow represents the DAPI positive fluorescence signal. The yellow arrow represents the TNF-α positive fluorescence signal. The white arrow represents the IL-6 positive fluorescence signal. Data are presented as mean ± SD (*n* = 8). Statistical significance was determined by an unpaired two-tailed Student’s *t*-test. * *p* < 0.05 versus the CON group.

To profile the inflammatory cascade triggered by IMD, a comprehensive assessment was conducted in carp hepatocytes (Figure 3). Transcriptional analysis via qRT-PCR revealed that IMD exposure induced a robust upregulation of key pro-inflammatory mediators (Figure 3a). Specifically, mRNA expression of TNF-α, IFN-γ, IL-1β, CXCL12, CCL2, IL-6, IL-18, and IL-23 was significantly elevated by 126.11%, 59.15%, 118.64%, 176.84%, 45.44%, 98.41%, 115.63%, and 160.93% (all *p* < 0.05), compared with CON group. Consistent with transcriptional alterations, IFN-γ, TNF-α, and IL-6 were substantially upregulated at the translational level, with protein abundances increasing by 99.31%, 41.43%, and 132.99%, respectively, in IMD-exposed hepatocytes (*p* < 0.05; Figure 3b). Furthermore, immunofluorescence staining for TNF-α and IL-6 provided corroborating evidence at the cellular level, demonstrating a pronounced enhancement in the fluorescence-positive area for TNF-α and IL-6 by 127.03% and 146.4%, respectively, following IMD treatment (*p* < 0.05; Figure 3c). Collectively, these multi-faceted data confirm that IMD exposure provokes a potent inflammatory response in carp hepatocytes, characterized by the coordinated induction of both cytokine and chemokine genes alongside their encoded proteins.

### 3.4. SQSTM1 Is a Direct Target of miR-153c in Hepatocytes Induced by IMD Exposure

**Figure 4:**
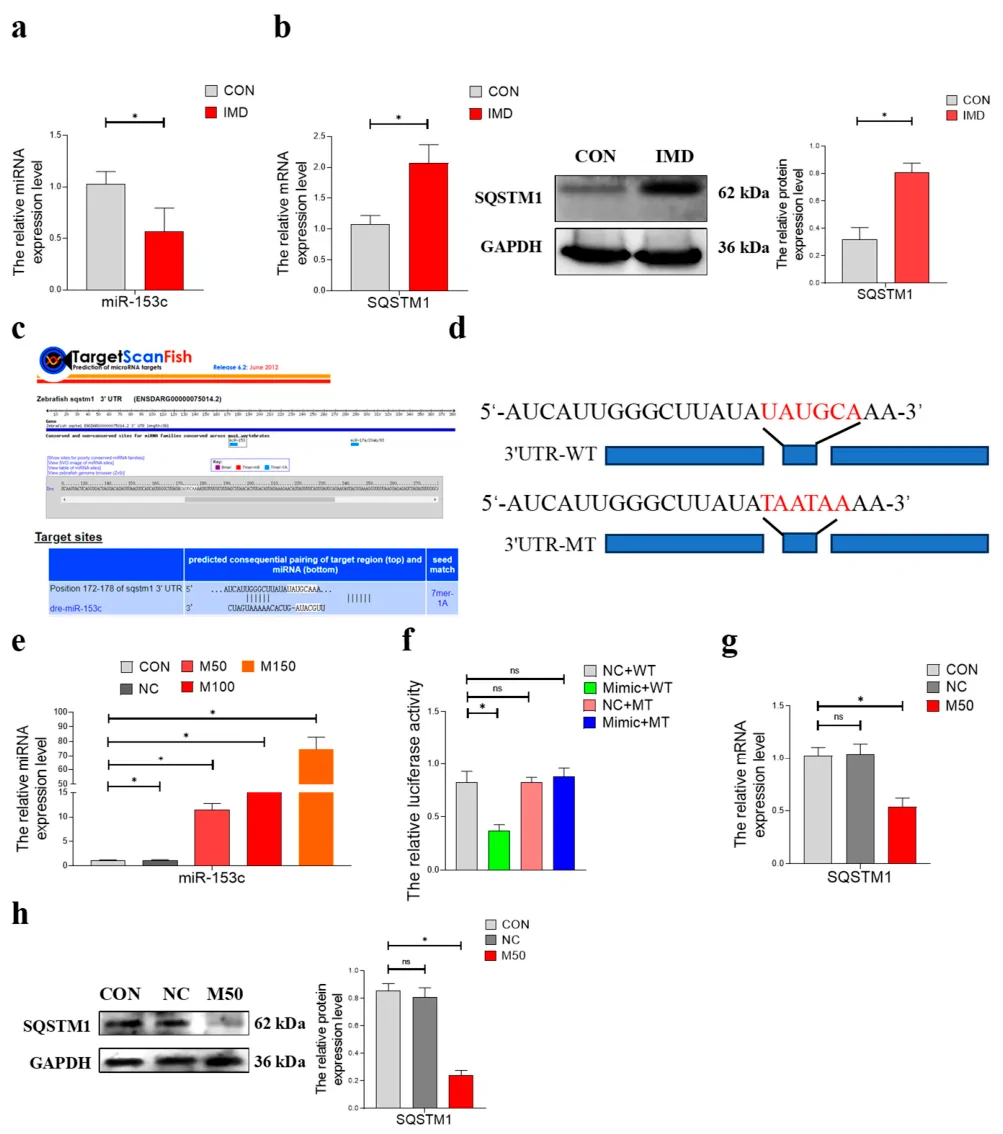
Direct targeting of SQSTM1 by miR-153c. (**a**) miR-153c levels were assessed via qRT-PCR in the CON and IMD groups (*n* = 8; *p* < 0.05). (**b**) SQSTM1 mRNA expression and GAPDH-normalized SQSTM1 protein abundance were measured after IMD treatment (*n* = 8; *p* < 0.05). (**c**,**d**) SQSTM1 was predicted to be a miR-153c target by TargetScanFish (**c**), with the sequence docking site illustrated (**d**) (*n* = 8; *p* < 0.05). (**e**) miR-153c levels in hepatocytes transfected with the indicated concentrations of miR-153c mimic (50, 100, 150 nM) (*n* = 8; *p* < 0.05). U6 was used as the reference. (**f**) Dual-luciferase reporter assay verifying direct targeting of the SQSTM1 3′-UTR by miR-153c. Carp primary hepatocytes were co-transfected with the wild-type (Luc-SQSTM1-WT) or seed-region mutant (Luc-SQSTM1-MUT) reporter together with miR-153c mimic or negative control (NC). Firefly luciferase activity was normalized to Renilla luciferase (*n* = 8; *p* < 0.05). (**g**,**h**) Transcript (**g**) and protein (**h**) abundances of SQSTM1 in hepatocytes from CON, NC and M50 (50 nM) groups (*n* = 8; *p* < 0.05). Data are presented as mean ± SD (*n* = 8). For panels (**a**,**b**), statistical significance was determined by an unpaired two-tailed Student’s *t*-test; * *p* < 0.05 versus the CON group. For panels (**e**–**h**), statistical significance was determined by one-way ANOVA followed by Tukey’s post hoc test; * *p* < 0.05 versus the CON group; “ns” indicates no significant difference (*p* ≥ 0.05).

To elucidate the underlying molecular mechanism, miR-153c expression was quantified via qRT-PCR, which revealed a 44.72% downregulation in the IMD group relative to the CON group (Figure 4a). qRT-PCR and WB analyses in hepatocytes verified increases of 48.3% and 60.1% in SQSTM1 mRNA and protein expression, respectively, in the IMD group (Figure 4b). TargetScanFish, a web-based predictive tool, identified SQSTM1 as a putative target of miR-153c (Figure 4c). Sequence alignment revealed a conserved recognition element within the 3′-UTR of SQSTM1 transcripts (Figure 4d). Collectively, these findings support SQSTM1 as a bona fide target of miR-153c in common carp (*Cyprinus carpio*) hepatocytes.

To further characterize miR-153c function, we conducted miR-153c overexpression assays to explore its functional role in carp (*Cyprinus carpio*) hepatocytes. As depicted in Figure 4e, qRT-PCR analysis confirmed a significant, dose-dependent upregulation of miR-153c in the overexpression groups. Compared to both the untreated CON and the negative control (NC) groups, the mRNA levels of miR-153c were markedly increased in the M50 (50 nM), M100 (100 nM), and M150 (150 nM) groups (*p* < 0.05). To validate whether SQSTM1 is a target gene of miR-153c, we constructed two dual-luciferase reporters carrying the wild-type and mutant SQSTM1 sequences. miR-153c was found to suppress luciferase activity of the SQSTM1 wild-type reporter. Notably, in contrast to the wild-type construct, the miR-153c mimic failed to inhibit luciferase activity mediated by the SQSTM1 mutant binding site in common carp (*Cyprinus carpio*) hepatocytes (Figure 4f). Using the established miR-153c mimic model (Figure 4g), miR-153c overexpression reduced SQSTM1 mRNA expression by 47.77% in the M50 group compared with the NC group. The protein level of SQSTM1 in the M50 group decreased by 71.68% compared with the CON group (Figure 4h). Collectively, these findings demonstrate that SQSTM1 serves as a specific downstream target of miR-153c in carp (*Cyprinus carpio*) hepatocytes.

### 3.5. The miR-153c Mimic Ameliorates Ferritinophagy in IMD-Exposed Hepatocytes by Regulating the miR-153c/SQSTM1 Axis

**Figure 5:**
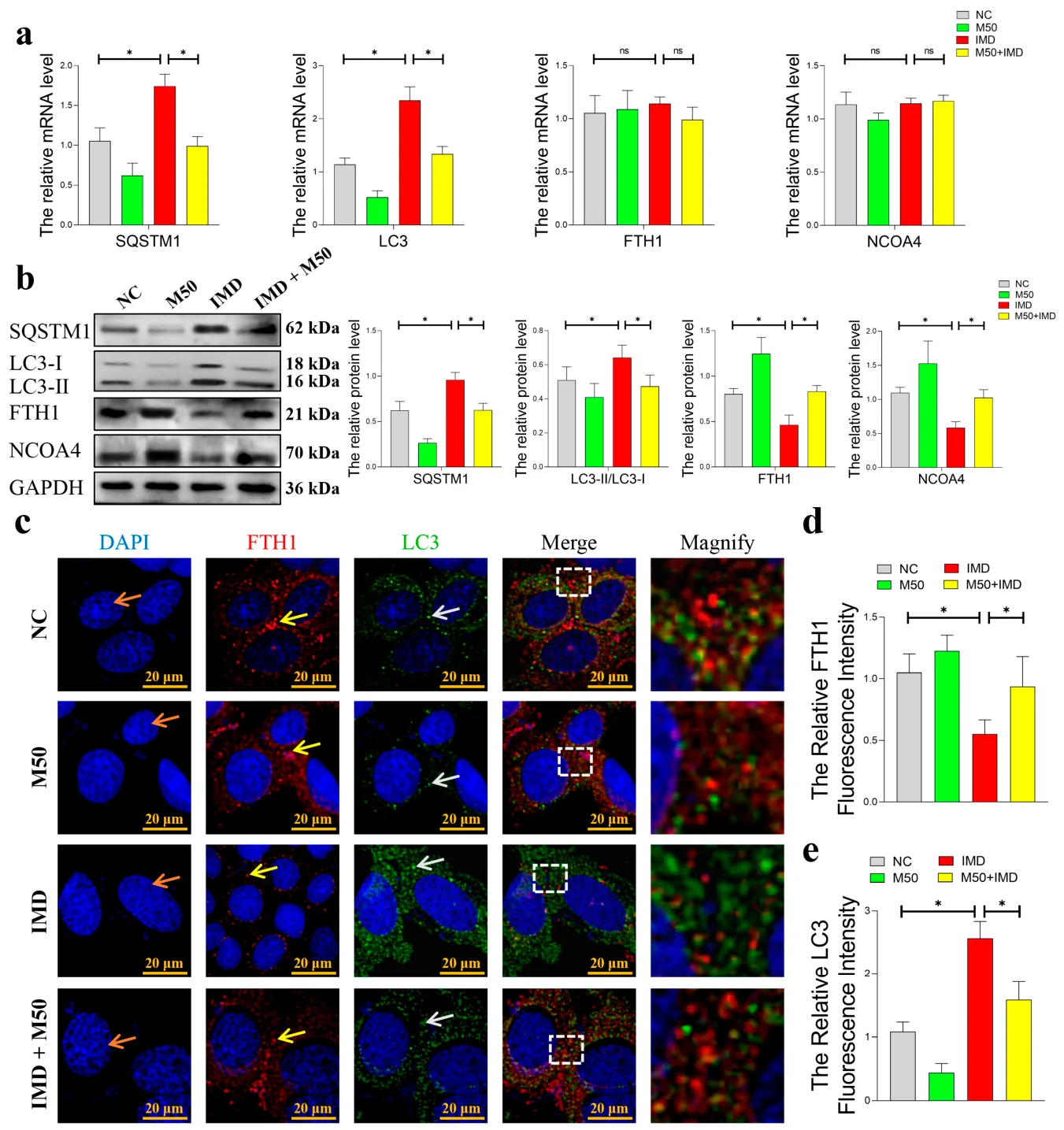
The 24-h IMD treatment alters miR-153c/SQSTM1-mediated ferritinophagy in hepatocytes. (**a**) The mRNA level of ferritinophagy in NC, M50, IMD and M50 + IMD groups (*n* = 8; *p* < 0.05). (**b**) The protein level of ferritinophagy in NC, M50, IMD and M50 + IMD groups (*n* = 8; *p* < 0.05). (**c**–**e**) Representative immunofluorescence images and quantification of FTH1 and LC3 fluorescence intensity in NC, M50, IMD and IMD + M50 groups (scale bars = 20 μm; mean ± SD, *n* = 8). The red arrow represents the DAPI positive fluorescence signal. The yellow arrow represents the FTH1 positive fluorescence signal. The white arrow represents the LC3 positive fluorescence signal (scale bars = 20 μm). Data are presented as mean ± SD (*n* = 8). Statistical significance was determined by one-way ANOVA followed by Tukey’s post hoc test. * *p* < 0.05 versus the NC group; “ns” indicates no significant difference (*p* ≥ 0.05). The white dashed square indicates the region of interest shown at higher magnification in the inset.

The impact of the miR-153c mimic on ferritinophagy was evaluated in IMD-treated hepatocytes. We hypothesized that ferritinophagy in hepatocytes responds to IMD treatment through regulation of miR-153c levels (Figure 5). As illustrated in Figure 5a, comparing IMD group to NC group, IMD group displayed elevated mRNA expression of SQSTM1 and LC3, increasing by 65.43% and 105.77% (*p* < 0.05). In contrast, no significant changes were observed in the mRNA levels of FTH1 and NCOA4. A comparison between IMD + M50 group and IMD group revealed decreased mRNA levels of SQSTM1 and LC3 (43.14% and 43.06%; *p* < 0.05). FTH1 and NCOA4 mRNA levels remained unchanged by the IMD + M50 intervention relative to the CON group. Figure 5b demonstrated that the protein levels of SQSTM1 and LC3-II/LC3-I increased substantially (by 54.09% and 25.96%; *p* < 0.05) in the IMD group versus NC group. Compared to NC group, IMD exposure significantly reduced the protein levels of FTH1 and NCOA4 by 42.41% and 46.68%, respectively. Relative to the IMD group, the IMD + M50 group exhibited marked decreases in SQSTM1 and LC3-II/LC3-I protein levels by 34.76% and 26.29%, respectively (*p* < 0.05). Furthermore, in comparison with IMD group, the protein levels of FTH1 and NCOA4 showed substantial increases of 79.37% and 75.27% in the IMD + M50 group, respectively (*p* < 0.05). Immunofluorescence staining for FTH1 and LC3 showed that the relative FTH1 fluorescence intensity was decreased by 47.46% in the IMD group compared with the NC group (Figure 5c–e). Compared with the IMD group, the IMD + M50 group exhibited a significant increase of 69.74% in FTH1 fluorescence intensity. Compared with the NC group, IMD treatment led to a 136.54% elevation in LC3 fluorescence intensity. Relative to the IMD + M50 group, LC3 fluorescence intensity was significantly increased by 38.09% in the IMD group. Importantly, these findings demonstrate that the miR-153c mimic counteracts ferritinophagy under IMD treatment through regulation of the miR-153c/SQSTM1 pathway in hepatocytes.

### 3.6. The miR-153c Mimic Ameliorates Ferroptosis and Inflammatory Responses in IMD-Exposed Hepatocytes by Regulating the miR-153c/SQSTM1 Axis

**Figure 6:**
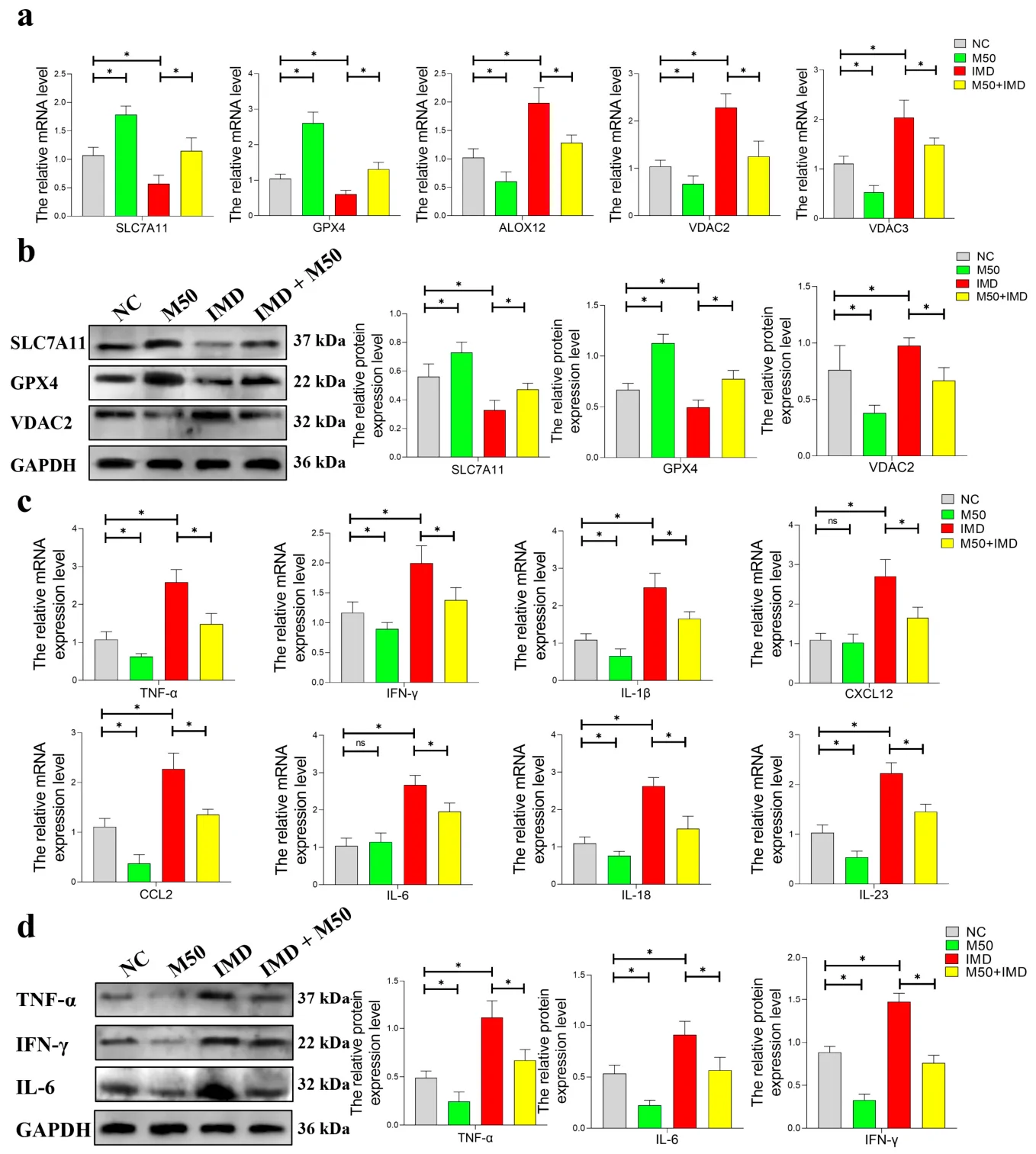
Effects of miR-153c treatment mimic on ferroptosis and inflammatory response in hepatocytes under exposure to IMD. (**a**) The mRNA levels of SLC7A11, GPX4, ALOX12, VDAC2 and VDAC3 in NC, M50, IMD and M50 + IMD groups (*n* = 8; *p* < 0.05). (**b**) GAPDH-normalized protein levels of ferroptosis-related markers in NC, M50, IMD and M50 + IMD groups (*n* = 8; *p* < 0.05). (**c**) The mRNA expression profiles of key inflammatory cytokines in NC, M50, IMD and M50 + IMD groups (*n* = 8; *p* < 0.05). (**d**) GAPDH-normalized protein abundances of cytokines in the NC, M50, IMD and M50 + IMD groups (*n* = 8; *p* < 0.05). Data are presented as mean ± SD (*n* = 8). Statistical significance was determined by one-way ANOVA followed by Tukey’s post hoc test. * *p* < 0.05 versus the NC group; “ns” indicates no significant difference (*p* ≥ 0.05).

To investigate the effect of the miR-153c/SQSTM1 pathway on IMD-induced ferroptosis and inflammatory responses in hepatocytes, carp hepatocytes were treated with the miR-153c mimic. We speculated that hepatocytes with low miR-153c levels undergo ferroptosis and inflammatory responses in response to IMD through SQSTM1 (Figure 6). SLC7A11 and GPX4 transcript levels were elevated by 101.44% and 116.79% (*p* < 0.05) in the IMD + M50 group relative to the IMD group alone (Figure 6a). Meanwhile, mRNA expression of ALOX12, VDAC2 and VDAC3 was suppressed by 35.29%, 45.25% and 26.9% (*p* < 0.05), respectively. The protein levels of SLC7A11 and GPX4 in the IMD group were significantly lower than those in the NC group by 41.59% and 25.9%, respectively, whereas the protein level of VDAC2 was 28.16% higher in the IMD + M50 group than in the IMD group (Figure 6b). miR-153c mimic + IMD intervention exhibited marked reductions in mRNA abundance of TNF-α, IFN-γ, IL-1β, CXCL12, CCL2, IL-6, IL-18 and IL-23, with decreases of 42.58%, 30.91%, 33.39%, 38.77%, 40.27%, 26.8%, 43.43% and 34.69% (*p* < 0.05) when compared with the IMD group (Figure 6c). The protein levels of TNF-α, IFN-γ, and IL-6 in the IMD group were higher than those in the NC group by 126.71%, 70.38%, and 65.96%, respectively, and lower in the IMD + M50 group than in the IMD group by 39.62%, 37.78%, and 48.25%, respectively (Figure 6d). Taken together, these results indicate that miR-153c mimic treatment partially, but not completely, reversed IMD-induced ferroptosis and inflammatory responses in carp (*Cyprinus carpio*) hepatocytes by regulating the miR-153c/SQSTM1 axis.

## 4. Discussion

This study delineates a comprehensive mechanistic pathway by which the widely used neonicotinoid insecticide IMD exerts hepatotoxicity in carp (*Cyprinus carpio*). We demonstrate that IMD exposure triggers a cascade of interconnected cellular events, culminating in hepatocyte death. The core sequence involves: (1) primary mitochondrial dysfunction (ATP, mtDNA, and NADPH/NADP^+^) and oxidative stress; (2) the activation of ferritinophagy, leading to Fe^2+^ overload; (3) the execution of ferroptosis as a dominant cell death modality; and (4) the instigation of a potent inflammatory response. Crucially, we identify the downregulation of miR-153c and its subsequent derepression of the selective autophagy receptor SQSTM1 as a critical upstream regulatory axis governing this toxicological pathway. Exogenous restoration of miR-153c effectively attenuated this cascade, revealing a potential therapeutic target for IMD-induced hepatotoxicity in carp.

The widespread use of IMD impacts aquatic organisms and contaminates aquatic ecosystems [27]. Studies have demonstrated that exposure to environmentally relevant concentrations of IMD elicits endocrine-disrupting effects and developmental toxicity in zebrafish [28]. The synergistic zebrafish neurodevelopmental toxicity of IMD is driven by mitochondrial dysfunction [29]. It has been documented that IMD exposure induced oxidative stress and inflammation in telencephalic zebrafish neurons, leading to reduced locomotor capacity and impaired social behaviors including heterosexual attraction and male defensive alertness [30]. Hepatic inflammation triggered by IMD exposure occurs through the activation of the NF-κB pathway [31]. In our study, IMD increased the mortality of hepatocytes isolated from common carp (*Cyprinus carpio*). Our preliminary cytotoxicity assessment confirmed the direct hepatotoxic effects of IMD on carp hepatocytes, with an IC_50_ value of 26.4 μM. This concentration-dependent decline in cell viability is consistent with and quantifies the hepatotoxic risk observed in vivo in other fish species exposed to IMD [31,32]. A concentration of 0.6 μM was selected for mechanistic investigations. This concentration, which approximates the upper range of IMD concentrations reported in contaminated surface waters (up to 157.5 μg/L, equivalent to approximately 0.62 μM), provides a sublethal stress model suitable for dissecting the sequential cellular events leading to cell death. It thereby extends the analysis beyond mere cytotoxicity assessments to explore the specific programmed cell death pathways involved. Nevertheless, additional mechanistic details underlying IMD-induced hepatotoxicity warrant further investigation.

Studies have demonstrated that exposure of zebrafish to the neonicotinoid pesticide IMD elicits excessive oxidative stress and DNA damage as key toxic outcomes [14]. IMD can induce mitochondrial dysfunction even at an extremely low concentration of 0.69 μM by disrupting mitochondrial membrane potential, depleting ATP levels, and perturbing calcium ion homeostasis, which in turn triggers oxidative stress and DNA damage [33]. These findings are consistent with our observation that the initial insult of IMD manifests at the mitochondrial level. The observed depletion of mtDNA, reduction in ATP, and a lowered NADPH/NADP^+^ ratio collectively indicate severe bioenergetic compromise and redox imbalance. This mitochondrial distress is further compounded by a shift in dynamics towards fission, as evidenced by the upregulation of Drp1 and Fis1. Mitochondria are both a major source and a key target of ROS; thus, their dysfunction directly explains the dramatic surge in ROS and the depletion of antioxidant enzymes (SOD and CAT). Our results corroborate and build upon earlier findings regarding pesticide-induced mitochondrial toxicity, positioning organellar failure as the foundational lesion in IMD hepatotoxicity. Such mitochondrial impairment is thus a probable key factor in *Cyprinus carpio* hepatocyte injury. To advance understanding, further research is needed to unravel the complex mechanistic basis of IMD-induced mitochondrial homeostasis disruption, identify the participating regulatory processes, and examine distinct molecular pathways.

Our findings reveal a profound alteration of the cytochrome P450 (CYP)-mediated xenobiotic metabolism pathway in IMD-exposed hepatocytes, which may reflect a broader cellular stress response and may contribute indirectly to intracellular oxidative imbalance. The majority of detected CYP isoforms (CYP2D6, CYP4M18, CYP6AE2, CYP9A32, CYP9A75, and CYP321A15) were significantly suppressed. This widespread downregulation aligns with studies showing that neonicotinoids, including IMD, can inhibit CYP enzyme activities in fish, impairing their primary detoxification capacity [34]. The suppression of these key metabolizing enzymes may be associated with impaired xenobiotic handling and prolonged cellular stress after IMD exposure. More importantly, although CYP enzymes are key players in redox homeostasis and xenobiotic metabolism, their catalytic cycles are also a source of endogenous ROS. The dysfunction of this system disrupts normal metabolic flux, potentially leading to the uncoupling of catalytic cycles and increased electron leakage, thereby contributing to the massive ROS burst we observed [35]. This pattern is consistent with a stress-response model in which excessive ROS and altered CYP expression may reinforce each other during IMD-induced cellular injury [36,37]. Conversely, the significant induction of CYP6AE44 and CYP6B4 presents a nuanced picture of metabolic adaptation or specific toxicant response [38]. Certain CYP subfamilies are known to be inducible by foreign compounds as part of a compensatory or stress-responsive mechanism. Their upregulation might represent an attempted adaptive response to metabolize the xenobiotic overload. However, this induction could be maladaptive. The increased expression of these specific isoforms might be associated with altered IMD metabolism and oxidative stress, although this possibility requires direct validation by CYP activity assays and metabolite profiling [39]. This selective induction amidst global suppression underscores a state of dysregulated metabolism, where the coordinated response of the detoxification network is shattered. This metabolic imbalance may contribute to the oxidative burden associated with mitochondrial dysfunction and lipid peroxidation, but the present data do not establish CYP dysregulation as a direct driver of ferroptosis. Thus, IMD-induced dysregulation of the ROS-xenobiotic metabolism pathway is best interpreted as a stress-associated change that may potentiate the oxidative environment permissive for ferroptosis, rather than as direct causal evidence for CYP-mediated ferroptosis execution.

The pivotal link between this mitochondrial damage and cell fate lies in the ferroptosis pathway [40]. The data presented here compellingly demonstrated that IMD exposure induced lipid peroxidation-driven ferroptosis in hepatocytes. The hallmark features of ferroptosis are unequivocally present: there is a profound dysregulation of iron homeostasis, with labile Fe^2+^ increasing over fourfold, and systematic dismantling of the cellular defense against phospholipid peroxidation. The coordinated downregulation of SLC7A11 and GPX4 at both mRNA and protein levels impairs the glutathione-dependent antioxidant system, rendering cells exquisitely vulnerable to oxidative damage [41]. Concurrently, the upregulation of pro-ferroptotic mediators such as ALOX12 (a lipoxygenase promoting lipid peroxidation) and VDAC2 (a mitochondrial pore protein linked to dysfunction) creates a cellular milieu poised for ferroptotic execution. This shift in the expression balance, favoring death signals over survival pathways, provides a clear molecular rationale for the observed cytotoxicity. We provide multifaceted evidence for ferroptosis: dysregulated iron homeostasis with massive Fe^2+^ accumulation; profound suppression of the system Xc^-^/GPX4 antioxidant axis (both transcriptionally and translationally); elevated MDA; and the upregulation of pro-ferroptotic mediators like ALOX12 and VDAC2. The depletion of SLC7A11 and GPX4, the central guardians against phospholipid peroxidation, is particularly consequential and marks a point of no return in the ferroptosis execution pathway.

Simultaneously, IMD triggers a potent and coordinated inflammatory response, characterized by the robust upregulation of a broad spectrum of pro-inflammatory cytokines (TNF-α, IL-1β, IL-6 and so on) and chemokines (CXCL12 and CCL2). Multi-level confirmation ranging from transcriptional activation to protein accumulation and cellular immunofluorescence underscores the strength and pervasiveness of this response. Critically, the induction of this specific cytokine profile, particularly IL-1β and IL-18, alongside the alarmin-like IFN-γ, provides a crucial mechanistic link to ferroptosis. Ferroptosis is now recognized as a highly immunogenic form of cell death [42]. The lipid peroxidation products and the release of DAMPs from damaged mitochondria during ferroptosis can directly activate pattern recognition receptors [43]. IMD-induced iron deposition and inflammation may further sensitize cells to iron-dependent cell death, ultimately driving the progression of hepatotoxicity.

Accumulating evidence indicates that ferritinophagy, a form of selective autophagy, serves as a critical initiator of ferroptosis by mediating iron overload, and microRNAs are key regulators of this autophagy–ferroptosis crosstalk in various toxicological processes [44]. Zhang et al. demonstrated that environmental toxicants can trigger ferritinophagy via disrupting miRNA-target gene networks, thereby promoting ferroptosis in hepatocytes [24]. Simone et al. further confirmed that SQSTM1, as a core autophagic adapter protein, was frequently targeted by miRNAs to modulate ferritinophagy through the SQSTM1/NCOA4 pathway, which in turn regulated intracellular iron homeostasis and lipid peroxidation [45]. Additionally, the crosstalk between ferroptosis and inflammatory responses is tightly controlled by miRNA-mediated signaling pathways, and inhibition of ferroptosis can effectively attenuate the activation of pro-inflammatory cytokines [46,47,48]. Importantly, in our experiment, we traced the source of the lethal iron surge to a ferritinophagy process in which SQSTM1 upregulation, secondary to miR-153c downregulation, promotes the autophagic delivery and degradation of FTH1 in cooperation with the canonical cargo receptor NCOA4. The liberation of sequestered iron fuels the Fenton reaction and exacerbates lipid peroxidation, thereby connecting autophagy dysregulation directly to ferroptotic death in carp hepatocytes exposed to IMD. Mechanistically, miR-153c was identified as a master regulator controlling the IMD-induced ferritinophagy–ferroptosis axis, and the conserved targeting of SQSTM1 mRNA by miR-153c, validated by luciferase reporter assays and miR-153c mimic overexpression experiments, establishes a clear causal relationship between miR-153c and SQSTM1 expression. Specifically, IMD-induced suppression of miR-153c relieves its post-transcriptional repression of SQSTM1, resulting in significant upregulation of SQSTM1 at both the mRNA (48.3%) and protein (60.1%) levels. The functional rescue experiments further confirm this regulatory axis: overexpression of the miR-153c mimic markedly reduces SQSTM1 expression, attenuates ferritinophagy as evidenced by partial recovery of FTH1 and NCOA4 protein levels and reduced LC3-II accumulation, partially restores GPX4 expression, and ultimately mitigates, rather than completely abolishes, ferroptosis in IMD-exposed hepatocytes, positioning the miR-153c/SQSTM1 node as a key regulatory switch in the IMD-induced hepatotoxicity pathway. The rescue effect was therefore interpreted as partial but biologically meaningful, because miR-153c overexpression reduced multiple IMD-induced injury markers by approximately 26.8% to 48.25% for inflammatory endpoints and substantially improved ferroptosis-related markers, but did not demonstrate complete normalization of hepatocyte status. Moreover, this study elucidates a consequential link between ferroptosis and inflammatory responses in this context, as evidenced by the robust upregulation of a broad spectrum of pro-inflammatory genes and chemokines (CXCL12 and CCL2) in IMD-exposed hepatocytes. These findings support the growing recognition of ferroptosis as an immunogenic process, which involves the release of DAMPs and lipid peroxidation products to activate innate immunity. This is consistent with the observed activation of multiple inflammasome and inflammatory signaling pathways. Notably, the miR-153c mimic not only rescued ferroptosis but also quelled this inflammatory response, suggesting that the inflammation observed in IMD-exposed hepatocytes is largely a downstream consequence of ferroptotic damage. These findings support the central role of the miR-153c/SQSTM1/ferritinophagy axis in IMD-induced hepatotoxicity in carp hepatocytes, while also raising the question of whether this stress-response pathway is conserved across aquatic species.

From an evolutionary and ecological perspective, the miR-153c/SQSTM1 axis may have broader relevance for aquatic toxicology. miRNAs are widely conserved post-transcriptional regulators in ray-finned fishes, and miR-153c has been annotated and functionally implicated in zebrafish, supporting its evolutionary conservation among teleosts [49,50]. SQSTM1/p62 is also a conserved selective autophagy receptor, and zebrafish studies have demonstrated that Sqstm1 participates in LC3-associated selective autophagy in vivo [51]. These conserved features suggest that the miR-153c/SQSTM1-mediated ferritinophagy response identified here may represent a stress-responsive module shared by multiple fish species. Nevertheless, miRNA-target interactions can evolve in a lineage-specific manner, and the direct regulation of SQSTM1 by miR-153c should not be assumed to be identical in all aquatic organisms. Therefore, our in vitro findings should be interpreted as mechanistic evidence in common carp hepatocytes rather than direct ecological proof at the organism or population level. Given that xenobiotic-induced oxidative stress and ferroptosis are increasingly recognized as important toxicological mechanisms in aquatic organisms [52,53], future studies should validate this pathway in vivo under environmentally relevant IMD exposure scenarios. Such studies should include chronic and pulsed exposure designs, multiple developmental stages, different fish species, tissue-level pathology, bioaccumulation, behavioral and reproductive endpoints, and rescue experiments targeting ferroptosis or SQSTM1-mediated ferritinophagy. These approaches will help determine whether the miR-153c/SQSTM1/ferritinophagy axis can serve as a conserved biomarker or intervention target for assessing neonicotinoid risks in aquatic ecosystems.

## 5. Conclusions

In conclusion, we have unraveled a coherent pathogenic circuit in which IMD silences miR-153c, leading to SQSTM1-mediated ferritinophagy. This disrupts iron storage, propelling carp hepatocytes into mitochondrial dysfunction, dysregulated CYP450 expression, ferroptosis, and inflammation. Overexpression of miR-153c, which inhibits the SQSTM1/ferritinophagy axis and ferroptosis, was accompanied by diminished inflammatory factor expression. This underscores the potential of miR-153c in reducing the cytotoxic effects of IMD on hepatocytes. This model deepens our insight into neonicotinoid toxicity and identifies the miR-153c/SQSTM1 interface as a potential conserved stress-response node for chemical-induced ferroptosis in fish liver, although its ecological relevance requires further validation in vivo and across aquatic species.

## Data Availability

The raw data supporting the conclusions of this article will be made available by the authors upon request.

## Supplementary Materials

The following supporting information can be downloaded at https://www.mdpi.com/article/10.3390/toxics14090814/s1, Table S1: The primers used in the present study; Table S2: The antibodies used in the present study; Table S3: Exact *p*-values for all statistical comparisons.
