# Supplementary material for: Imidacloprid-Induced Ferroptosis-Associated Injury in Common Carp Hepatocytes: Potential Involvement of the miR-153c/SQSTM1 Axis
Source: Toxics. 2026 Sep 13;14(9):814. doi: 10.3390/toxics14090814 (PMC13611238; doi:10.3390/toxics14090814)
Supplement: Supplementary file 1 [file toxics-14-00814-s001.zip › toxics-4489128-supplementary.pdf]

Supplementary Material

Imidacloprid-Induced Ferroptosis-Associated Injury in Common Carp Hepatocytes: Potential Involvement of the miR-153c/SQSTM1 Axis

Huijie Chen [1], Yitong Wang [1], Zhenkai Yao [2], Jing Li [3], Peng Li [1] and Lei Diao [1,*]

[1] School of Biological and Pharmaceutical Engineering, Jilin Agricultural Science and Technology College, 77 Hanlin Road, Jilin 132101, China
[2] Vaccine Inspection Department, Jilin Provincial Drug Inspection and Verification Center, No. 657 Zhanjiang Road, Economic and Technological Development Zone, Changchun 130033, China
[3] Drug and Cosmetics Inspection Laboratory, Inspection and Testing Center, Jiyuan Industry-City Integration Demonstration Zone, No. 181 North Wenchang Road, Jiyuan 459000, China
* Correspondence: diaolei@jlnku.edu.cn; Tel.: +86-451-5519-0407

Supplementary Tables

Table S1 The primers used in the present study

Gene	Forward primer (5'-3')	Reverse primer (5'-3')
SLC7A11	CGCCATTGCCATTATCATTGTTCCT	AACCAGCCAGCATAAGCATACAT
GPX4	AGTAAGATTGATGTGAACGGTGACT	TGATGCCATTTCCAAAGATTCCT
Ferritin	AGTGTTAGAACCAGCGCAGT	TCTGAGCATGTTCGCGTTCT
ALOX12	CCACAACCACTCTGAATGA	ACATCTTAGCCAACAACCA
VDAC2	GGATATGGATTCGGTATGGT	GCTTGTATGCGGTCTTGA
VDAC3	GTTAATGATGGCACAGAGTT	TTAGCAGAGACGGAGGAA
Fis1	CAGACATAGTAGCACCAGAA	TCCAATACCACCGACAATC
Drp1	ATCGCTGCTGAACTCCTA	TGCCACAATGCTGAACAA
OPA1	CTACAGAAGGCACTACTACAT	CCAGAACAGAACCACATCA
Mfn1	CGTGAAGAATATAGTGGATGAG	TGATGGAGCAGGATGGAA
TFAM	TAATCTGCTGGTCAAGTCAT	TTGCCTGCGTAGTAGTTC
TNF-α	ACCAGGCTTTCACTTCAGGC	TAGCCGCCATAGGAATCGGA
IFN-γ	TGTGCCCGAGAACCTAGACA	AGGATTCGCAGGAAGATGGG
IL-1β	CTCCGCTCCACATCTCGTACTC	CATCTCCACCATCTGCGAATCTTC
CXCL12	CCTTAGCCTGGTGGTCAT	GCCTTGTCAATGTAAGTCTTC
CCL2	GTCTGTCTGGTGCTCTTC	ATGCGTCGTATGATAACTCT
IL-6	CGGTCCACTCGATCCTGTTC	CCTCTTGGGGTCTTTCCCTC
IL-23	CCCAAGGACTCAGGGACAAC	TGGAGGCTGCGAAGGATTTT
IL-18	AAGAGCGGAATAAGGAGCAATGC	TGCTTACAAGGTGATGTCACAGA
CYP2D6	TGCCAGAATGTTGGAGGACC	GGAGGATGATCCCAACGAGG
CYP4M18	AGAGGACCGAATACATTGAC	CTCGCTGTAGTATCGTGAC
CYP6AE2	GAGAACCGTGGCTACAAG	ATCAATAAGGCGTCGTCAA

CYP9A32	TCTTCGGCAACCTCTTCT	ACACGCTTCAACATCTCC
CYP9A75	ACTATGACGCTGCTCTTG	AGTTGCTGTATGGAGTTGTA
CYP321A15	ATACGGAACACTTACAACCT	AGCAGCGAAGAAGAACAA
CYP6AE44	TATGGCGACAAGTGGAAG	GCTCTCATTCTGGCAGTAT
CYP6B4	AGAACCAGAGCAGCAGAT	ATATCCAGCGGCGTAGAA
SQSTM1	TGCTACTGCCTCTTCTCA	GTCCATTCCTCATCACTACA
LC3	GTGTTGTCGTCAAGGTAGT	AGTGAGGTTGTGGAATGTG
FTH1	CGTGAAGAAACCAGAGAAGGATGAG	TATGAGGGTCGTTGTGCTGAGAG
NCOA4	ATCTGGAGGAGGTGGAAGTGGAAG	TTTATCTTGCTTGACCGGGCTTGG
GAPDH	AGTCGGAGTCAACGGATT	CTGAGATGATAACACGCTTAG
miR-153c	GCGCAUCAUUGGGCUUAUAUA	-
U6	CACGCAAATTCGTGAAGCGTTCCA	-

Table S2 The antibodies used in the present study

Antibody name	Dilution ratio	Company information
SQSTM1	1:3500	Wanleibio, China
LC3	1:1000	Wanleibio, China
NCOA4	1:1000	Wanleibio, China
FTH1	1:3000	Sigma, USA
SLC7A11	1:3000	Sigma, USA
GPX4	1:2000	Wanleibio, China
VDAC2	1:3000	Wanleibio, China
TNF-α	1:1500	Wanleibio, China
IFN-γ	1:3000	Santa Cruz, USA
IL-6	1:3000	Wanleibio, China
GAPDH	1:20000	Sigma, USA

Table S3. Exact P-values for all statistical comparisons

Figure	Panel	Measurement	Comparison	Test	Exact P-value
Fig. 1	1a	Cell viability (0.6 μM)	CON vs IMD	t-test	$P < 0.05$
Fig. 1	1a	Cell viability (1 μM)	CON vs IMD	t-test	$P < 0.05$
Fig. 1	1a	Cell viability (10 μM)	CON vs IMD	t-test	$P < 0.05$
Fig. 1	1a	Cell viability (25 μM)	CON vs IMD	t-test	$P < 0.05$
Fig. 1	1a	Cell viability (50 μM)	CON vs IMD	t-test	$P < 0.05$
Fig. 1	1a	Cell viability (100 μM)	CON vs IMD	t-test	$P < 0.05$
Fig. 1	1a	Cell viability (200 μM)	CON vs IMD	t-test	$P < 0.05$
Fig. 1	1b	Intracellular Fe^{2+}	CON vs IMD	t-test	$P < 0.05$
Fig. 1	1c	SLC7A11 mRNA	CON vs IMD	t-test	P = 0.0059
Fig. 1	1c	GPX4 mRNA	CON vs IMD	t-test	P = 0.0033
Fig. 1	1c	Ferritin mRNA	CON vs IMD	t-test	$P < 0.05$
Fig. 1	1c	ALOX12 mRNA	CON vs IMD	t-test	P = 0.0340
Fig. 1	1c	VDAC2 mRNA	CON vs IMD	t-test	P = 0.0150
Fig. 1	1c	VDAC3 mRNA	CON vs IMD	t-test	P = 0.0284
Fig. 1	1d	SLC7A11 protein	CON vs IMD	t-test	$P < 0.05$
Fig. 1	1d	GPX4 protein	CON vs IMD	t-test	$P < 0.05$
Fig. 1	1d	VDAC2 protein	CON vs IMD	t-test	$P < 0.05$
Fig. 1	1e	ATP content	CON vs IMD	t-test	$P < 0.05$
Fig. 1	1f	mtDNA content	CON vs IMD	t-test	P = 0.0228
Fig. 1	1g	NADPH/$NADP^+$ ratio	CON vs IMD	t-test	P = 0.0186
Fig. 1	1h	Fis1 mRNA	CON vs IMD	t-test	P = 0.0060
Fig. 1	1h	Drp1 mRNA	CON vs IMD	t-test	$P < 0.05$
Fig. 1	1h	OPA1 mRNA	CON vs IMD	t-test	P = 0.0303

Fig. 1	1h	Mfn1 mRNA	CON vs IMD	t-test	P = 0.0069
Fig. 1	1h	TFAM mRNA	CON vs IMD	t-test	P = 0.0033
Fig. 2	2a	Intracellular ROS	CON vs IMD	t-test	P = 0.0183
Fig. 2	2b	MDA content	CON vs IMD	t-test	P < 0.05
Fig. 2	2b	T-AOC	CON vs IMD	t-test	P < 0.05
Fig. 2	2b	CAT activity	CON vs IMD	t-test	P < 0.05
Fig. 2	2b	SOD activity	CON vs IMD	t-test	P < 0.05
Fig. 2	2c	CYP2D6 mRNA	CON vs IMD	t-test	P < 0.05
Fig. 2	2c	CYP4M18 mRNA	CON vs IMD	t-test	P < 0.05
Fig. 2	2c	CYP6AE2 mRNA	CON vs IMD	t-test	P < 0.05
Fig. 2	2c	CYP9A32 mRNA	CON vs IMD	t-test	P < 0.05
Fig. 2	2c	CYP9A75 mRNA	CON vs IMD	t-test	P < 0.05
Fig. 2	2c	CYP321A15 mRNA	CON vs IMD	t-test	P < 0.05
Fig. 2	2c	CYP6AE44 mRNA	CON vs IMD	t-test	P < 0.05
Fig. 2	2c	CYP6B4 mRNA	CON vs IMD	t-test	P < 0.05
Fig. 3	3a	TNF-α mRNA	CON vs IMD	t-test	P < 0.05
Fig. 3	3a	IFN-γ mRNA	CON vs IMD	t-test	P < 0.05
Fig. 3	3a	IL-1β mRNA	CON vs IMD	t-test	P < 0.05
Fig. 3	3a	CXCL12 mRNA	CON vs IMD	t-test	P < 0.05
Fig. 3	3a	CCL2 mRNA	CON vs IMD	t-test	P < 0.05
Fig. 3	3a	IL-6 mRNA	CON vs IMD	t-test	P < 0.05
Fig. 3	3a	IL-18 mRNA	CON vs IMD	t-test	P < 0.05
Fig. 3	3a	IL-23 mRNA	CON vs IMD	t-test	P < 0.05
Fig. 3	3b	IFN-γ protein	CON vs IMD	t-test	P < 0.05
Fig. 3	3b	TNF-α protein	CON vs IMD	t-test	P < 0.05
Fig. 3	3b	IL-6 protein	CON vs IMD	t-test	P < 0.05

Fig. 3	3c	TNF-α fluorescence	CON vs IMD	t-test	P < 0.05
Fig. 3	3c	IL-6 fluorescence	CON vs IMD	t-test	P < 0.05
Fig. 4	4a	miR-153c level	CON vs IMD	t-test	P < 0.05
Fig. 4	4b	SQSTM1 mRNA	CON vs IMD	t-test	P < 0.05
Fig. 4	4b	SQSTM1 protein	CON vs IMD	t-test	P < 0.05
Fig. 4	4e	miR-153c (50 nM)	CON vs M50	ANOVA/Tukey	P < 0.05
Fig. 4	4e	miR-153c (100 nM)	CON vs M100	ANOVA/Tukey	P < 0.05
Fig. 4	4e	miR-153c (150 nM)	CON vs M150	ANOVA/Tukey	P < 0.05
Fig. 4	4f	Luc-SQSTM1-WT	WT+NC vs WT+mimic	ANOVA/Tukey	P = 0.0000202
Fig. 4	4f	Luc-SQSTM1-MUT	MUT+NC vs MUT+mimic	ANOVA/Tukey	P = 1.0000 (NS)
Fig. 4	4g	SQSTM1 mRNA	NC vs M50	ANOVA/Tukey	P < 0.05
Fig. 4	4h	SQSTM1 protein	NC vs M50	ANOVA/Tukey	P < 0.05
Fig. 5	5a	SQSTM1 mRNA	NC vs IMD	ANOVA/Tukey	P < 0.05
Fig. 5	5a	SQSTM1 mRNA	IMD vs IMD+M50	ANOVA/Tukey	P < 0.05
Fig. 5	5a	LC3 mRNA	NC vs IMD	ANOVA/Tukey	P < 0.05
Fig. 5	5a	LC3 mRNA	IMD vs IMD+M50	ANOVA/Tukey	P < 0.05
Fig. 5	5b	SQSTM1 protein	NC vs IMD	ANOVA/Tukey	P < 0.05
Fig. 5	5b	SQSTM1 protein	IMD vs IMD+M50	ANOVA/Tukey	P < 0.05
Fig. 5	5b	LC3-II/LC3-I protein	NC vs IMD	ANOVA/Tukey	P < 0.05
Fig. 5	5b	LC3-II/LC3-I protein	IMD vs IMD+M50	ANOVA/Tukey	P < 0.05
Fig. 5	5b	FTH1 protein	NC vs IMD	ANOVA/Tukey	P = 0.0027

Fig. 5	5b	FTH1 protein	IMD vs IMD+M50	ANOVA/Tukey	P = 0.0110
Fig. 5	5b	NCOA4 protein	NC vs IMD	ANOVA/Tukey	P < 0.05
Fig. 5	5b	NCOA4 protein	IMD vs IMD+M50	ANOVA/Tukey	P < 0.05
Fig. 5	5c–e	FTH1 fluorescence	NC vs IMD	ANOVA/Tukey	P < 0.05
Fig. 5	5c–e	FTH1 fluorescence	IMD vs IMD+M50	ANOVA/Tukey	P < 0.05
Fig. 5	5c–e	LC3 fluorescence	NC vs IMD	ANOVA/Tukey	P < 0.05
Fig. 5	5c–e	LC3 fluorescence	IMD vs IMD+M50	ANOVA/Tukey	P < 0.05
Fig. 6	6a	SLC7A11 mRNA	IMD vs IMD+M50	ANOVA/Tukey	P < 0.05
Fig. 6	6a	GPX4 mRNA	IMD vs IMD+M50	ANOVA/Tukey	P < 0.05
Fig. 6	6a	ALOX12 mRNA	IMD vs IMD+M50	ANOVA/Tukey	P < 0.05
Fig. 6	6a	VDAC2 mRNA	IMD vs IMD+M50	ANOVA/Tukey	P < 0.05
Fig. 6	6a	VDAC3 mRNA	IMD vs IMD+M50	ANOVA/Tukey	P < 0.05
Fig. 6	6b	SLC7A11 protein	NC vs IMD	ANOVA/Tukey	P < 0.05
Fig. 6	6b	GPX4 protein	NC vs IMD	ANOVA/Tukey	P < 0.05
Fig. 6	6b	VDAC2 protein	IMD vs IMD+M50	ANOVA/Tukey	P < 0.05
Fig. 6	6c	TNF-α mRNA	IMD vs IMD+M50	ANOVA/Tukey	P < 0.05
Fig. 6	6c	IFN-γ mRNA	IMD vs IMD+M50	ANOVA/Tukey	P < 0.05

Fig. 6	6c	IL-1β mRNA	IMD vs IMD+M50	ANOVA/Tukey	$P < 0.05$
Fig. 6	6c	CXCL12 mRNA	IMD vs IMD+M50	ANOVA/Tukey	$P < 0.05$
Fig. 6	6c	CCL2 mRNA	IMD vs IMD+M50	ANOVA/Tukey	$P < 0.05$
Fig. 6	6c	IL-6 mRNA	IMD vs IMD+M50	ANOVA/Tukey	$P < 0.05$
Fig. 6	6c	IL-18 mRNA	IMD vs IMD+M50	ANOVA/Tukey	$P < 0.05$
Fig. 6	6c	IL-23 mRNA	IMD vs IMD+M50	ANOVA/Tukey	$P < 0.05$
Fig. 6	6d	TNF-α protein	NC vs IMD	ANOVA/Tukey	$P < 0.05$
Fig. 6	6d	TNF-α protein	IMD vs IMD+M50	ANOVA/Tukey	$P < 0.05$
Fig. 6	6d	IFN-γ protein	NC vs IMD	ANOVA/Tukey	$P < 0.05$
Fig. 6	6d	IFN-γ protein	IMD vs IMD+M50	ANOVA/Tukey	$P < 0.05$
Fig. 6	6d	IL-6 protein	NC vs IMD	ANOVA/Tukey	$P < 0.05$
Fig. 6	6d	IL-6 protein	IMD vs IMD+M50	ANOVA/Tukey	$P < 0.05$

Data are presented as mean ± SD (n = 8). Two-group comparisons were analyzed by an unpaired two-tailed Student's t-test; multi-group comparisons were analyzed by one-way ANOVA followed by Tukey's post hoc test.
